# Identification and detection of a novel point mutation in the Chitin Synthase gene of *Culex pipiens* associated with diflubenzuron resistance

**DOI:** 10.1371/journal.pntd.0008284

**Published:** 2020-05-01

**Authors:** Emmanouil A. Fotakis, Valentina Mastrantonio, Linda Grigoraki, Daniele Porretta, Arianna Puggioli, Alexandra Chaskopoulou, Hugo Osório, Mylène Weill, Romeo Bellini, Sandra Urbanelli, John Vontas

**Affiliations:** 1 Department of Crop Science, Pesticide Science Lab, Agricultural University of Athens, Athens, Greece; 2 Institute of Molecular Biology and Biotechnology, Foundation for Research and Technology Hellas, Heraklion, Crete, Greece; 3 Department of Environmental Biology, Sapienza University of Rome, Rome, Italy; 4 Vector Biology Dept, Liverpool School of Tropical Medicine, Liverpool, United Kingdom; 5 Medical and Veterinary Entomology Dept., Centro Agricoltura Ambiente "G. Nicoli", Bologna, Italy; 6 European Biological Control Laboratory, USDA-ARS, Thessaloniki, Greece; 7 National Institute of Health Doctor Ricardo Jorge, Centro de Estudos de Vetores e Doenças Infeciosas Doutor Francisco Cambournac, Avenida da Liberdade, Águas de Moura, Portugal; 8 Institut des Sciences de l’Evolution (CNRS-Université de Montpellier-IRD-EPHE), Montpellier, France; Fundaçao Oswaldo Cruz, BRAZIL

## Abstract

**Background:**

Diflubenzuron (DFB) is one of the most used insecticides in mosquito larval control including that of *Culex pipiens*, the proven vector of the recent West Nile Virus epidemics in Europe. Two mutations (I1043L and I1043M) in the chitin synthase (*CHS*) putative binding site of DFB have been previously reported in *Cx*. *pipiens* from Italy and associated with high levels of resistance against this larvicide.

**Methodology/Principal findings:**

Here we report the identification of a third mutation at the same I1043 position of the CHS gene resulting in the substitution of Isoleucine to Phenylalanine (I1043F). This mutation has also been found in agricultural pests and has been functionally validated with genome editing in *Drosophila*, showing to confer striking levels (>15,000 fold) of DFB resistance. The frequency of the I1043F mutation was found to be substantially higher in *Cx*. *pipiens* mosquitoes surviving DFB doses largely exceeding the recommended field dose, raising concerns about the future efficient use of this insecticide. We monitored the presence and frequency of DFB mutations in *Cx*. *pipiens* mosquitoes from several Mediterranean countries, including Italy, France, Greece, Portugal and Israel. Among the *Cx*. *pipiens* populations collected in Northern Italy all but one had at least one of the three DFB mutations at allele frequencies reaching 93.3% for the I1043M, 64.8% for the I1043L and 10% for the I1043F. The newly reported I1043F mutation was also identified in two heterozygote individuals from France (4.2% allelic frequency). In contrast to Italy and France, no DFB resistant mutations were identified in the *Cx*. *pipiens* mosquitoes sampled from Greece, Portugal and Israel.

**Conclusions/Significance:**

The findings of our study are of major concern for mosquito control programs in Europe, that rely on the use of a limited number of available larvicides, and highlight the necessity for the development of appropriate Insecticide Resistance Management (IRM) programs, to ensure the sustainable use of DFB.

## Author summary

Diflubenzuron is one of the main larvicides used for the control of the West Nile Virus vector *Culex pipiens* in the Mediterranean. However, the efficiency of control is now under threat due to the selection of insecticide resistance. Two point mutations were previously identified at the Chitin synthase and shown to confer low and high levels of resistance and a diagnostic was developed to monitor the trait. This study reports the identification of a third mutation associated with high levels of diflubenzuron resistance in Italy. This mutation was also detected in France, whereas no resistance mutations were found in *Cx*. *pipiens* mosquitoes sampled from Greece, Portugal and Israel. The findings are of major concern for mosquito control programs in S. Europe, which rely on the use of a limited number of larvicides.

## Introduction

Mosquito and vector borne disease (VBD) control largely rely on the use of chemical insecticides. Larviciding, targeting immature stage mosquitoes, is pivotal for managing nuisance and vector populations, and yet relies on a small number of available insecticides. Diflubenzuron (DFB) is among the most important larvicides used against mosquitoes, especially in Europe, where under the current European Union (EU) regulations, neurotoxic insecticides such as temephos are prohibited for use in mosquito breeding sites. DFB is a chitin synthesis inhibitor and belongs to the Benzoyl(phenyl)urea family (BPUs-Group 15 based on the IRAC grouping system). It inhibits the chitin biosynthesis process and causes abortive molting [1] by directly interacting with the chitin synthase 1 (CHS1) enzyme which is responsible for chitin synthesis in the insect cuticle [2].

In Europe, over the last decades DFB has been used extensively in agriculture and forestry pest control and more recently also in mosquito control, being one of the main larvicides used for reducing the population size of the primary West Nile virus (WNV) vector *Culex pipiens* [3]. *Cx*. *pipiens* is amongst the most abundant mosquito species in the Mediterranean region and, apart from a major nuisance problem, it is the proven vector of the recent WNV epidemics/outbreaks in Europe [4,5] which makes its control a public health priority. *Cx*. *pipiens* is also a world-wide concern as it transmits several more diseases including lymphatic filariasis, Japanese encephalitis, Saint Louis encephalitis and Rift valley fever causing mortality and morbidity.

Selection of insecticide resistance in vector species and agricultural pests, due to the extensive use of a limited number of insecticides in public health and agriculture, is a major problem seriously impeding vector control efforts across the globe [6].

DFB resistance was detected for the first time in *Cx*. *pipiens* specimens sampled in 2015 from Ravenna (Italy) [7]. The highly resistant phenotypes recorded were associated with two point mutations at the 1043 amino acid of the Chitin synthase 1 (*CpCHS1*) gene; I1043M and I1043L. The susceptible individuals carry a gene sequence corresponding to the Isoleucine amino acid (named as “I1043” hereafter), while the mutant individuals carry sequences corresponding to the Leucine and/or Methionine amino acids (hereafter named as “I1043L” and “I1043M” respectively).

The introduction of the two mutations in *Drosophila melanogaster* (CHS gene) with the genome editing method CRISPR/Cas9 showed that they both confer significant levels of resistance: the I1043M mutation (I1056M in *Drosophila*) results in a Resistance Ratio of >15.000 folds and the I1043L (I1056L in *Drosophila*) mutation in a Resistance Ratio of >20 folds [2,7].

A third mutation at the I1043 site of the chitin synthase gene resulting in a substitution from Isoleucine to Phenylalanine (hereafter named as “I1043F”) has been reported in the major agricultural pest *Plutella xylostella* (I1017F) and associated with resistance to DFB [8]. This mutation was also functionally validated with CRIPSR/Cas9 in *D*. *melanogaster* (I1056F in *Drosophila*) in exactly the same way as the other two reported mutations and was shown to confer >15,000 fold resistance to DFB [2]. This mutation has not been found yet in mosquitoes.

A PCR-RFLP diagnostic assay and an allele specific PCR assay have been developed [7] to identify the I1043M and I1043L mutations respectively and in 2017 the distribution and frequency of the two DFB mutations was recorded in *Cx*. *pipiens* populations from the Emilia Romagna region, Northern Italy [3]. The mutated allele I1043L was detected in 20 out of the 30 *Cx*. *pipiens* populations at an allele frequency ranging from 4 to 60%, while the I1043M mutation was detected in 10 out of the 30 populations at a frequency ranging from 8 to 77.1%. Mutation presence and distribution was focal (distinctly higher mutation frequencies were recorded in the Eastern provinces) and associated with the history of agricultural and mosquito DFB applications.

High DFB mutation (I1043L, I1043M) frequencies, reaching a frequency of 52.7% were recently also reported in *Cx*. *pipiens* populations sampled in 2016 from Mugla province, Western Turkey [9]. On the contrary, no resistant mutations were recorded in specimens analyzed from Greece and France [3]. The findings from Italy and Turkey are alarming and of major concern for public health, highlighting the need for systematic DFB resistance monitoring in vector populations in Europe, an essential prerequisite for the design and implementation of appropriate insecticide resistance management strategies.

Here, we report the detection of a third DFB resistance mutation—I1043F, in the CHS1 gene of *Cx*. *pipiens* and monitor the presence and geographical distribution of all three mutations in *Cx*. *pipiens* populations from the Mediterranean basin using direct sequencing.

## Methods

### Study sites, samples and mosquito handling

*Cx*. *pipiens* specimens were collected during the summer of 2018 from the Eastern provinces of Emilia-Romagna (Northern Italy). Field caught mosquitoes non-exposed to a bioassay, as well as survivors from bioassays with DFB collected in 2017 from Cervia were included in the analysis. Samples from Greece (collected from three regions between 2014–2017), France (2011), Portugal (2018–2019) and Israel (2010) were also included in the analysis (Table in S1 Table). All collections were conducted on public land.

Adult samples were collected with CDC light traps baited with dry ice, while larvae dipping collections were conducted for larvae sampling. In all larvae sampling events, larvae were collected from at least five breeding sites in each locality, so as to reduce the probability of collecting and analyzing siblings for DFB resistance. The 3rd-4th instar larvae and adult mosquitoes were identified morphologically to species [10] and stored in ethanol 90%.

### DNA extraction

Genomic DNA was extracted from single *Cx*. *pipiens* larvae and adult specimens using the C-TAB method [11]. According to this method, mosquitoes were crushed with a plastic pestle in a 1.5 ml microcentrifuge tube containing 200μl of extraction buffer previously heated at 60°C (5% CTAB,1.4 M NaCl, 0.2% 2-b mercaptoethanol, 20 mM EDTA, 100 mMTris-HCl pH 8.0). Then, each tube was incubated at 60°C for 10 min and proteins were removed with one volume chloroform/isoamyl alcohol (24:1). DNA was precipitated with one volume of isopropanol and the pellet was washed with 70% ethanol. Finally, the DNA was re-suspended in 30μl of double distilled water and preserved at -20°C. The concentration and quality of the extracted DNA was measured for each sample using NanoDrop ND-1000 (Thermo Scientific, DE, USA).

### Amplification and analysis of the chitin synthase sequences

A 825 bp fragment of the CHS gene spanning the site 1043 was amplified in the individual specimens using the primers CHSseqF (5’-CCGCGTTCAAGATTGACAACTGG-3’) and CHSseqR (5’TCCAGTAGGGGTTCGTCAGG-3’) [7]. The PCR reaction was carried out in a total volume of 25μl containing 5ng DNA, 2.5 μl of 10X buffer, 0.4 mM dNTPs, 0.4 μM primers and 2U of the DreamTaq Green DNA Polymerase (Thermo Scientific, USA). The PCR conditions were as follows: 95°C for 5 min, 30 cycles of 94°C for 30 sec, 60°C for 30 sec, 72°C for 1 min and a final extension of 72°C for 10 min. 5 ul of the reaction were analyzed by electrophoresis on a 1% agarose gel to verify successful amplification and the remaining 20 ul were purified using a PCR purification kit (Macherey-Nagel) and sent for sequencing (Microsynth AG, Switzerland and Cemia, Greece), (Sanger sequencing / platform: 3730xl DNA analyser), using the primer CP_kkv_R1 5′- ACGTTTGCGGGTGTGATGTC-3′ [7]. The sequences obtained were analyzed with the software program BioEdit 7.2 (Biological Sequence Alignment Editor). Samples with poor quality sequencing were excluded from the genotype/allele frequency analyses.

## Results

### Identification of a novel diflubenzuron resistance mutation in *Culex pipiens*

**Fig 1:**
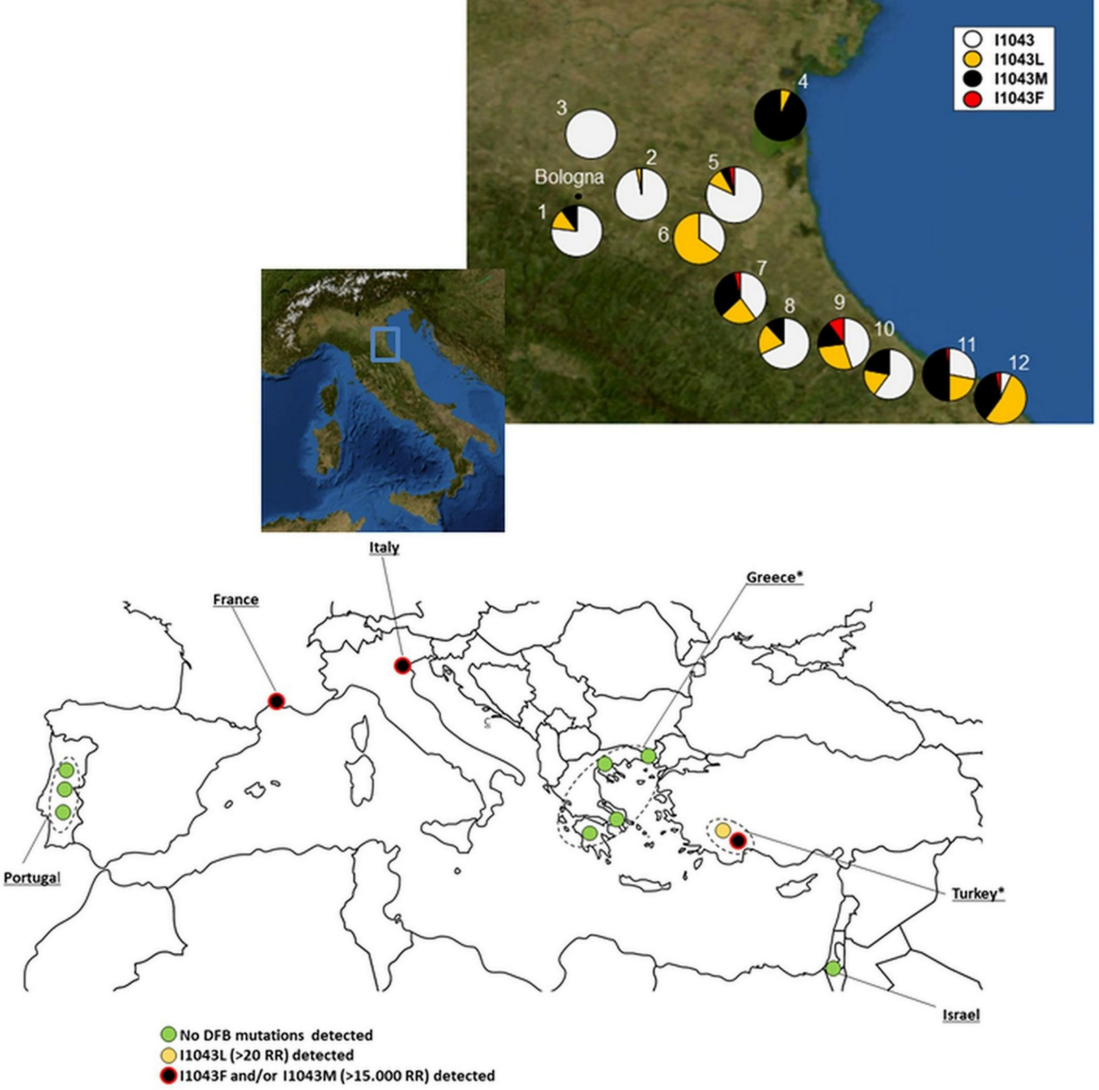
Distribution of DFB resistance mutations in *Cx*. *pipiens* populations in the Mediterranean basin (bottom) and relative frequencies of the susceptible and resistant alleles found in populations of *Cx*. *pipiens* collected from the Emilia-Romagna provinces, Italy (2018) (top)—(map images were obtained from [12]). Sampled localities in Italy are numbered according to Table 1. * DFB resistance mutation analyses in *Cx*. *pipiens* populations from the Peloponnese, Greece and Mugla Province, Turkey were conducted in [13] and [9], respectively.

A sequence of 825 bp, part of the chitin synthase C-terminus, the putative binding site of DFB, spanning the 1043 position (*Cx*. *pipiens* numbering) was amplified from *Cx*. *pipiens* gDNA (Italy, 2018 samples) and examined for the presence of mutations. The sequencing results showed a high reliability of this approach in discriminating susceptible and resistant genotypes, as shown in S1 Fig. In addition to the previously reported I1043L and I1043M DFB resistance mutations in *Cx*. *pipiens*, the mutation I1043F that has been associated with very high levels of DFB resistance in *Pl*. *xylostella*, was found in the analysed samples (Table 1; Fig 1).

**Table 1:** Detection and monitoring of CHS alleles associated with DFB resistance in *Cx*. *pipens* populations from Italy sampled in 2018.

Locality		Genotype frequency										Allele frequency (%)			
	N	II	IL	LL	IM	MM	LM	FF	IF	FL	FM	(I)	(L)	(M)	(F)
1. Bologna (BO)	30	0.60	0.20	0.03	0.14	0.03	--	--	--	--	--	76.6	13.3	10	0
2. Budrio (BO)	30	0.93	0.07	--	--	--	--	--	--	--	--	96.6	3.3	0	0
3. Rubizzano (BO)	28	1.0	--	--	--	--	--	--	--	--	--	100	0	0	0
4. Lido delle Nazioni (FE)	30	--	--	--	--	0.87	0.13	--	--	--	--	0	6.6	93.3	0
5. Lugo (RA)	30	0.70	0.20	--	0.03	--	--	--	--	--	0.07	81.6	10	5	3.3
6. Massa Lombarda (RA)	27	0.30	0.11	0.59	--	--	--	--	--	--	--	35.1	64.8	0	0
7. Forlì (FC)	30	0.17	0.23	--	0.23	0.13	0.17	--	--	0.07	--	40	23.3	33.3	3.3
8. Bertinoro (FC)	30	0.46	0.30	--	0.14	--	0.10	--	--	--	--	68.3	20	11.6	0
9. Cesena (FC)	30	0.23	0.37	0.07	0.03	0.03	0.07	--	0.03	--	0.17	45	28.3	16.6	10
10. Santarcangelo di Romagna (RN)	30	0.30	0.23	0.03	0.37	--	0.07	--	--	--	--	60	18.3	21.6	0
11. Rimini (RN)	30	0.12	0.03	--	0.30	0.17	0.35	--	--	0.03	--	28.3	21.6	48.3	1.6
12. Riccione (RN)	30	--	0.10	0.17	0.03	0.07	0.56	--	--	0.07	--	6.6	53.3	36.6	3.3

Frequency of each CHS-1043 genotype in the Italian populations analysed and the corresponding allele frequencies. The number given to each locality matches the number on Fig 1. N = total number of individuals analysed. I = wild susceptible allele I1043; L = resistant allele I1043L; M = resistant allele I1043M; F = resistant allele I1043F. The abbreviation in brackets refers to province. BO: Bologna, FE: Ferrara; RA: Ravenna, FC: Forlì-Cesena, RN: Riccione

This mutation has been functionally characterised and shown to confirm very high levels of DFB resistance [2]. We subsequently re-analysed by sequencing samples collected from Italy in 2017 (Cervia population), including survivors from high dosage DFB bioassays. As shown in Table 2, this third mutation was already present in 2017, at a low frequency (5,5%) in the population. In line with the I1043M resistance mutation, which also gives a very high resistance phenotype and was found substantially enriched in survivors of DFB bioassays ([7] and Table 2), the frequency of the I1043F resistance mutation also increased substantially (from 5.5% to 14.29%) in the bioassay survivors (0.468 ppm) of the Cervia population (Table 2). All survivors from the high DFB dose were either homozygotes for the I1043M mutation or heterozygotes having a combination of two mutations (I1043M/I1043F and I1043L/I1043F) (Table 2), while none had the susceptible allelic form, not even in a heterozygote state.

**Table 2:** Number and frequency of chitin synthase alleles in high DFB dose bioassay survivors compared to mosquitoes from the same population.

Population	Na	Genotypes							
		I1043 (RR = 1)		1043L (RR >20x)		1043M (RR> 15.000x)		1043F (RR> 15.000x)	
	Alleles	%	Alleles	%	Alleles	%	Alleles	%
Cervia– 2017	**74**	40	54,05	14	18,91	16	21,62	4	5,41
Cervia–2017, survivors	**14**	-	0	1	7,14	11	78,57	2	14,29

Na = total number of alleles; survivors = individuals that survived a diflubenzuron dose of 0,468 ppm. R.R = The Resistance Ratio to diflubenzuron conferred by each allele (in homozygosity) when introduced with CRISPR/Cas9 in *Drosophila melanogaster*. The frequency of mutated alleles was significantly higher (p-value<0.0001, Fisher’s test) in bioassay survivors.

### Frequency of diflubenzuron resistance mutations in field populations from Italy

To monitor the presence and frequency of DFB resistance mutations in *Cx*. *pipiens* mosquitoes from Italy we analysed 355 individuals from 12 localities collected in 2018 by direct sequencing. The sequencing results showed the presence of the previously reported I1043L and I1043M mutations, but also the presence of the novel I1043F mutation in the populations from Italy. One population (Rubizzano) showed only susceptible alleles (i.e. I1043) (Table 1, Fig 1). In the remaining 11 populations, the occurrence of resistant alleles was observed. The I1043F mutation was found in 5 out of the 12 populations analysed. In particular, it was present in mosquitoes collected in the provinces of Ravenna, Forlì-Cesena and Riccione, where it was found at an allele frequency ranging from 1.6–10% (Table 1, Fig 1). In all tested populations, the I1043F allele was never found in homozygosity, but only in heterozygous genotypes, with either the susceptible or one of the other two mutated alleles (i.e. I1043L and I1043M) (Table 1, Fig 1).

The other two mutations, I1043L and I1043M were identified in the tested populations and in some cases at a very high frequency. The I1043L resistant allele was found in 11 populations, with an allele frequency ranging from 3.3–64.8%. The I1043M resistant allele was found in 9 populations and its frequency ranged from 11.6–93.3%. The co-occurrence of the I1043L and I1043M resistant alleles was observed in 9 populations and in 7 out of them heterozygous individuals I1043L/I1043M were found with a frequency ranging from 0.07 to 0.56 (genotype frequency). The occurrence of all three mutations was found in 5 out of the 12 populations analysed (Table 1, Fig 1).

### Monitoring of DFB resistance mutations in *Cx*. *pipiens* field populations from other Mediterranean countries

*Cx*. *pipiens* mosquitoes were collected from four countries: Greece, France, Portugal and Israel and analysed individually for detection of the DFB resistant mutations I1043L, I1043M and I1043F. The mutation I1043F was found at a low frequency in France (Region of Montpellier). Two individuals out of the 24 tested (4.2% allelic frequency) were heterozygous for this mutation (I1043/I1043F), while none of the other two mutations was identified.

A total of 91 mosquitoes were analyzed from Greece. All individuals were homozygous for the susceptible allele I1043. Similarly to Greece, no resistance mutations were detected in *Cx*. *pipiens* mosquitoes analysed from Portugal (N = 23) and Israel (N = 11) (all samples were homozygous for the susceptible allele I1043).

## Discussion

Two Chitin synthase mutations, I1043M and I1043L have been previously reported in *Cx*. *pipiens* mosquitoes, the major vector of West Nile virus, and associated with high levels of resistance to diflubenzuron [7]. Here we report the presence of a third Chitin synthase mutation, the I1043F in *Cx*. *pipiens* populations from Northern Italy and France (Montpellier-Southern France). The presence of the novel mutation was correlated with the DFB resistance phenotype in *Cx*. *pipiens*, as its allelic frequency increased substantially in bioassay survivors of high DFB doses. The I1043F mutation has been previously associated with DFB resistance in the major agricultural pest *Plutella xylostella* [2], and it has been functionally characterized by CRISPR/Cas9 in *Drosophila*, showing to confer very high levels of resistance against DFB (>15,000-fold), similar to the I1056M mutation [2], and substantially higher than the I1043L (20-fold) [7]. *Cx*. *pipiens* individuals either homozygous for the mutation I1043M or heterozygotes for mutations I1043L/I1043F or I1043M/I1043F survived at DFB concentrations exceeding the recommended WHO dose (i.e. 0.25 ppm of DFB, under optimum spraying conditions), which indicates that this resistance can dramatically affect DFB performance in the field. Indeed, this has already been observed following quality control treatment, regularly conducted in the frame of the Emilia-Romagna, (Northern Italy) mosquito control plan. The findings are of major concern for public health, as DFB is used in many places for the control of *Cx*. *pipiens* and *Aedes* arbovirus vectors and it is in fact one of the very few active ingredients available in Europe, where neurotoxic insecticides have been banned from use in mosquito breeding sites (EU Biocides Regulation 528/2012). The diagnostic assays (PCR-RFLP and Allele specific PCR) developed by [7] have been used successfully to identify DFB resistance mutations in *Cx*. *pipiens* and are recommended as a monitoring tool. However, they are unable to detect the I1043F mutation. The described PCR-RFLP assay detects specifically the presence of the I1043M allele as a NlaIII restriction site is created by the mutation (CATC → CAT**G**). The allele specific PCR uses internal primers that hybridize preferentially to the susceptible allele and the allele coding for the I1043L mutation ATC→CTC substitution. Thus, it is predicted that the PCR bands generated for a homozygous individual for the I1043F mutation would only have the common 352bp band, generated by the external primers, while a heterozygote I1043/I1043F would have the same pattern as a homozygous susceptible individual. This highlights the dangers of advocating a single diagnostic test for detecting insecticide resistance [14]. In this study we sequenced a part of the *CHS* gene in *Cx*. *pipiens* individuals collected in twelve localities in Italy (with an intense history of DFB applications and reported presence of the DFB mutations I1043M/1043L [3]). We identified in all localities but one the presence of at least one of the three DFB resistance mutations. In five populations, the frequency of the resistance alleles reached levels higher than 50%, while extreme cases were also observed where the susceptible allele was not identified. Among the three *CpCHS1* mutations, the newly reported I1043F was found at an allele frequency ranging from 1.6–10%, while the other two mutations were generally found at higher frequencies ranging from 10–93.3% for I1043M and 3.3–64.8% for I1043L. The lower allelic frequency observed for I1043F in the tested populations could be due to a random difference in the selection of the three mutations or related to potential differences in their fitness costs [2]. However, this remains to be tested. The selection of resistance to DFB in Italy seems to be associated with the extensive use of this active ingredient over the past twenty-thirty years against agricultural pests, also potentially driven by the use of DFB against mosquitoes over the last ten-fifteen years. The presence and distribution of DFB resistance mutations appear to be focal, primarily towards the Eastern Emilia-Romagna provinces in Italy. This was proposed to be related to the presence of a significantly higher number of orchards in the region resulting in a heavier use of DFB for agricultural purposes and a higher selective pressure to the local *Cx*. *pipiens* populations [3]. Contrary to Italy, DFB resistance was not detected in *Cx*. *pipiens* mosquitoes sampled from Greece (despite the intensive use of this product as a larvicide for more than a decade), Israel and Portugal possibly due to different selection pressure regimes imposed on these populations. Nevertheless, the novel DFB resistance mutation I1043F was also found in Montpellier France, so far at low frequencies (4.2% allelic frequency). A recent study [9] has reported the presence of mutations I1043L and I1043M in *Cx*. *pipiens* mosquitoes collected in the Turkish province of Muğla. Mutation I1043M was identified at an allelic frequency ranging from 25% to 52.7% and mutation I1043L at a frequency ranging from 15.7% to 37.5%. Again, the extensive use of DFB for both mosquito and agricultural pest control in the region were hypothesized to be the driving force for the selection of resistance and the presence of the associated *CHS* mutations at high frequencies. Our results are of major concern for public health in Europe. DFB is an important insecticide used for controlling arbovirus mosquito vectors. It is not only used to control *Culex* mosquitoes, as for example in Europe to prevent WNV outbreaks, but also in other countries, like in Latin America, where it is applied in drinking water to control dengue transmitting *Ae*. *aegypti* mosquitoes [15]. The insurgence of DFB resistance in *Cx*. *pipiens* populations in Italy and potentially elsewhere, given that mutations have already been found in France and Turkey [9], in combination with the limited availability of alternative insecticides highlights the necessity for the development of appropriate Insecticide Resistance Management (IRM) programs, to ensure the sustainability of current control interventions. Rotating between other larvicide active ingredients, as well as incorporating other vector control methodologies [16] in parallel to larviciding could help in managing the current situation. Ongoing insecticide resistance surveillance in Europe for *Cx*. *pipiens* and other vector species such as *Ae*. *albopictus* are prerequisites for the development and execution of IRM strategies resulting in efficient and effective arbovirus vector control. In addition, concerns regarding the reduction of reliable biocides for routine and emergency mosquito control, as in the case of epidemics or invasion of new mosquito species, should reinforce the development of additional mosquito larvicides and possibly the re-consideration of old active ingredients.

## Supporting information

S1 Fig Chromatograms showing susceptible (II) and resistant genotypes (LL, IL, IM, MM, FL, FM) found in the *Cx*. *pipiens* individuals analysed. The site where the mutations occur in the CHS gene is highlighted with a black circle.
(TIF)

S1 Table Study site details. (DOCX)

## References

[1] Merzendorfer H. Chitin synthesis inhibitors: old molecules and new developments. Insect Sci. 2013;20(2):121–38. 10.1111/j.1744-7917.2012.01535.x 23955853

[2] Douris V, Steinbach D, Panteleri R, Livadaras I, Pickett JA, Van Leeuwen T, et al Resistance mutation conserved between insects and mites unravels the benzoylurea insecticide mode of action on chitin biosynthesis. Proc Natl Acad Sci U S A. 2016;113(51):14692–7. 10.1073/pnas.1618258113 27930336 PMC5187681

[3] Porretta D, Fotakis EA, Mastrantonio V, Chaskopoulou A, Michaelakis A, Kioulos I, et al Focal distribution of diflubenzuron resistance mutations in Culex pipiens mosquitoes from Northern Italy. Acta Trop. 2019;193:106–12. 10.1016/j.actatropica.2019.02.024 30825446

[4] Chaskopoulou A, L'Ambert G, Petric D, Bellini R, Zgomba M, Groen TA, et al Ecology of West Nile virus across four European countries: review of weather profiles, vector population dynamics and vector control response. Parasit Vectors. 2016;9(1):482 10.1186/s13071-016-1736-6 27590848 PMC5009705

[5] Gomes B, Kioulos E, Papa A, Almeida AP, Vontas J, Pinto J. Distribution and hybridization of Culex pipiens forms in Greece during the West Nile virus outbreak of 2010. Infect Genet Evol. 2013;16:218–25. 10.1016/j.meegid.2013.02.006 23466890

[6] Paine M.J.I., Brooke B. Insecticide Resistance and Its Impact on Vector Control. In: Horowitz A., Ishaaya I. (eds) Advances in Insect Control and Resistance Management Springer, Cham; 2016 pp 287–312.

[7] Grigoraki L, Puggioli A, Mavridis K, Douris V, Montanari M, Bellini R, et al Striking diflubenzuron resistance in Culex pipiens, the prime vector of West Nile Virus (vol 7, 11699, 2017). Sci Rep-Uk. 2018;8. 10.1038/s41598-017-12103-1 PMC5601912 28916816

[8] Van Leeuwen T, Demaeght P, Osborne EJ, Dermauw W, Gohlke S, Nauen R, et al Population bulk segregant mapping uncovers resistance mutations and the mode of action of a chitin synthesis inhibitor in arthropods. Proc Natl Acad Sci U S A. 2012;109(12):4407–12. 10.1073/pnas.1200068109 22393009 PMC3311382

[9] Guz N, Çağatay N.S, Fotakis E.A, Durmuşoğlu E, Vontas J. Detection of diflubenzuron and pyrethroid resistance mutations in Culex pipiens from Muğla, Turkey. Acta Trop. 2020; 203:105294 10.1016/j.actatropica.2019.105294 31836282

[10] Schaffner F, Angel G, Geoffroy B, Hervy J, Rhaiem A, Brunhes J. The Mosquitoes of Europe/Les Moustiques d’Europe. Logiciel d’identification Et d’enseignement (CD-Rom). IRD Editions & EID MeÂditerrane Âe, Montpellier, France 2001.

[11] Navajas M, Lagnel J, Fauvel G, de Moraes G. Sequence variation of ribosomal internal transcribed spacers (ITS) in commercially important Phytoseiidae mites. Exp Appl Acarol. 1999;23(11):851–9. 10.1023/a:1006251220052 10668860

[12] Stamen Design; 2020 [cited 2020 Apr 20]. Map images [Internet]. Available from http://maps.stamen.com/#terrain/12/37.7706/-122.3782

[13] Mavridis K, Fotakis EA, Kioulos I, Mpellou S, Konstantas S, Varela E, et al Detection of West Nile Virus—Lineage 2 in Culex pipiens mosquitoes, associated with disease outbreak in Greece, 2017. Acta Trop. 2018;182:64–8. 10.1016/j.actatropica.2018.02.024 29474832

[14] Vontas J, Mavridis K. Vector population monitoring tools for insecticide resistance management: Myth or fact? Pestic Biochem Physiol. 2019;161:54–60. 10.1016/j.pestbp.2019.08.005 31685197

[15] Belinato TA, Valle D. The Impact of Selection with Diflubenzuron, a Chitin Synthesis Inhibitor, on the Fitness of Two Brazilian Aedes aegypti Field Populations. PLOS ONE. 2015; 10(6):e0130719 10.1371/journal.pone.0130719 26107715 PMC4481264

[16] Achee NL, Grieco JP, Vatandoost H, Seixas G, Pinto J, Ching-Ng L, et al Alternative strategies for mosquito-borne arbovirus control. PLoS Negl Trop Dis. 2019;13(1):e0006822 10.1371/journal.pntd.0006822 30605475 PMC6317787

