# Supplementary figures and images for: Identification and detection of a novel point mutation in the Chitin Synthase gene of Culex pipiens associated with diflubenzuron resistance
Source: PLoS Negl Trop Dis. 2020 May 1;14(5):e0008284. doi: 10.1371/journal.pntd.0008284 (PMC7219787; doi:10.1371/journal.pntd.0008284)

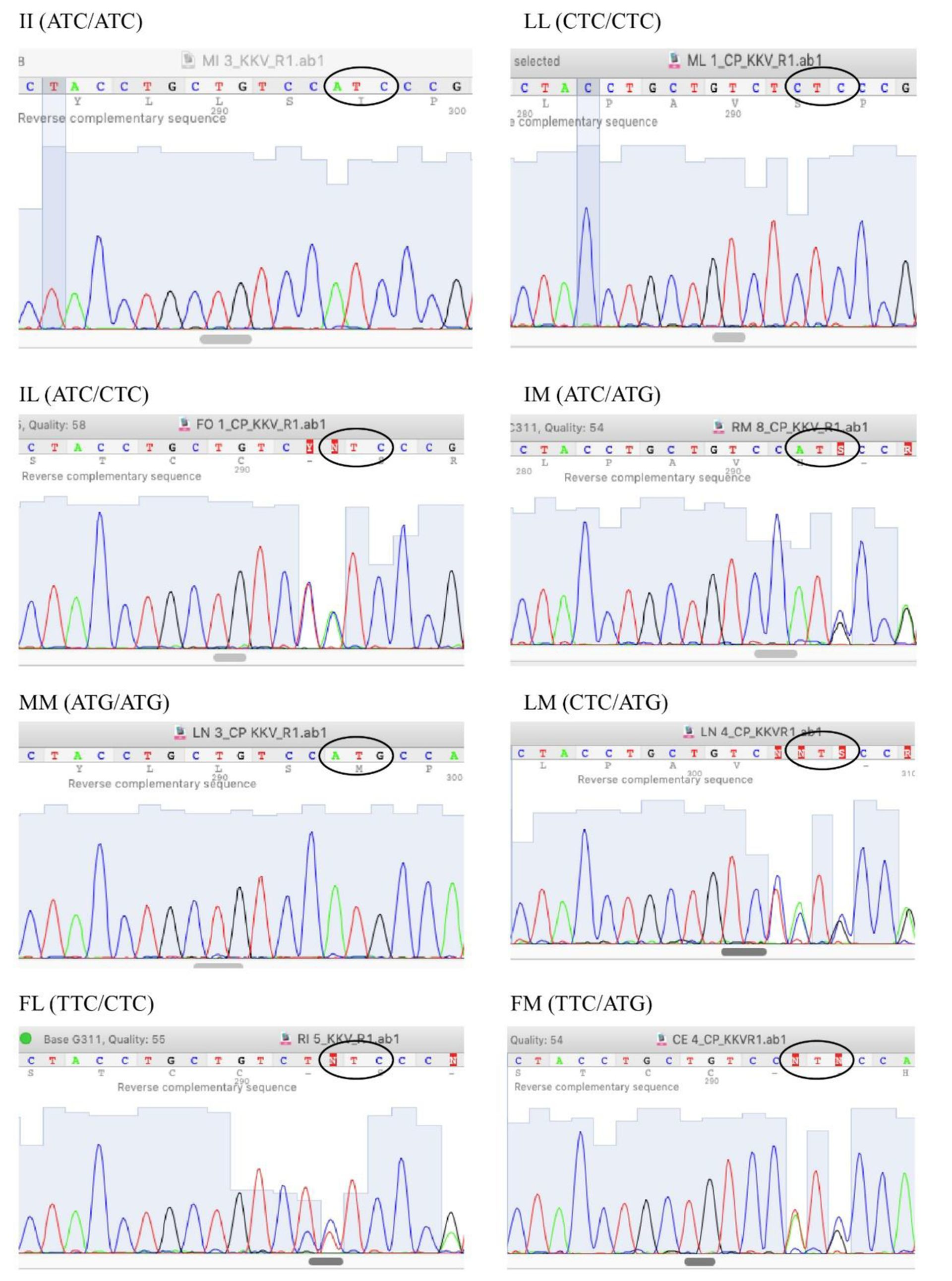

Supplement: S1 Fig — The site where the mutations occur in the CHS gene is highlighted with a black circle. (TIF) [file pntd.0008284.s001.tif]
