# Supplementary material for: Identification and detection of a novel point mutation in the Chitin Synthase gene of Culex pipiens associated with diflubenzuron resistance
Source: PLoS Negl Trop Dis. 2020 May 1;14(5):e0008284. doi: 10.1371/journal.pntd.0008284 (PMC7219787; doi:10.1371/journal.pntd.0008284)
Supplement: S1 Table — (DOCX) [file pntd.0008284.s002.docx]

**Supplementary Table 1. Study site details**

| **Country** | **Region** | **Field sites (N)** | **Site characteristics** | **Collection year** | **Analyzed specimens (N*)** |
| --- | --- | --- | --- | --- | --- |
| Italy | Northern Italy / Eastern Emilia Romagna | 12 | Urban / peri-urban | 2018 | 355 |
|  | Northern Italy / Ravenna | 1 | Peri-urban | 2017 | 88 |
| France | Southern France / Occitanie | 1 | Agricultural | 2011 | 24 |
| Portugal | Central Portugal / Coimbra | 2 | Agricultural | 2018 | 12 |
|  | Southern Portugal / Alentejo | 2 | Agricultural | 2019 | 11 |
| Greece | Northern Greece / Thessaloniki R.U | 2 | Agricultural | 2014 | 20 |
|  | Northern Greece / Thessaloniki R.U | 2 | Agricultural | 2017 | 20 |
|  | Northern Greece / Evros R.U | 2 | Agricultural | 2014 | 25 |
|  | Central Greece / Attica R.U | 2 | Urban | 2015 | 26 |
| Israel | Gush Dan | 1 | Urban | 2010 | 11 |

(N) is the number of field sites and (N*) the number of specimens analyzed per region. Field site specific locations: Israel: Tel Aviv; Greece: Thessaloniki, Evros, Athens; Portugal: Condeixa-a-Nova, Mértola, Beja, Grândola; France: Montpellier; Italy: all sites are listed in Table 1. R.U = Regional Unit. The field sites were selected based on: the history of DFB applications (Italy and Greece sites), previous findings regarding DFB mutation(s) presence [3,7] and the availability of *Culex* samples (mosquito collections / surveillance programs).
